# Relationship between ground reaction force and sacrum acceleration during 180° change of direction maneuvers in elite female basketball players

**DOI:** 10.3389/fspor.2026.1665797

**Published:** 2026-02-12

**Authors:** Hiroki Ogata, Daichi Yamashita, Naoto Nishikawa, Toshiharu Yokozawa, Masako Hoshikawa

**Affiliations:** 1 Graduate School of Physical Education, National Institute of Fitness and Sports in Kanoya, Kagoshima, Japan; 2 Japan Basketball Association, Tokyo, Japan; 3 Japan Institute of Sports Sciences, Japan High Performance Sports Center, Tokyo, Japan

**Keywords:** 180° turn, lateral shuffle, cutting, agility, kinetics, inertial measurement unit

## Abstract

**Background:**

This study investigated the relationship between sacrum acceleration (ACC_IMU_) measured using an inertial measurement unit (IMU) and ground reaction force (GRF) measured using force plates during 180° change of direction (COD) maneuvers in elite female basketball players.

**Methods:**

Fourteen Japanese national female basketball players performed two types of 180° COD tasks (lateral shuffle and 180° turn maneuvers) on force plates while wearing a sacrum-mounted IMU, completing two trials in each direction (left and right). The peak horizontal GRF during plant foot contact was normalized to body weight (GRF_rel_), whereas peak horizontal and resultant ACC_IMU_ were expressed relative to gravitational acceleration, allowing direct comparison between dimensionless quantities.

**Results:**

Reliability across the two trials was assessed using intraclass correlation coefficients (ICC_2,2_) and coefficients of variation (CV), and was acceptable for most variables (ICC_2,2_ = 0.67–0.95; CV% = 3.85–12.74%). Paired *t*-tests revealed that peak horizontal ACC_IMU_ was significantly greater than peak horizontal GRF_rel_ across all conditions (*p* < 0.001, *d* = 0.56–1.06). Pearson correlation and ordinary least products regression analyses demonstrated a significant association between peak horizontal ACC_IMU_ and peak horizontal GRF_rel_ during the lateral shuffle (*r* = 0.55–0.69, *p* < 0.05), with the ACC_IMU_ increasing proportionally to the GRF_rel_ (slope = 4.55–5.23), but not during the 180° turn (*r* = 0.33–0.49, *p* > 0.05). Peak resultant ACC_IMU_ was significantly correlated with peak horizontal GRF_rel_ (*r* = 0.64–0.72, *p* < 0.05) and exhibited proportional bias (slope = 2.61–4.70).

**Conclusion:**

These results indicate that, despite potential software-related errors and estimation uncertainties, ACC_IMU_ monitoring represents a promising method for estimating peak horizontal GRF_rel_ demands during task-specific 180° COD maneuvers in real-world settings.

## 1 Introduction

Basketball is a sport that frequently demands rapid change-of-direction (COD) movements, often executed in small spaces (1). Time-motion analyses indicate that players perform several hundred to over one thousand discrete movements during a single game, characterized by frequent directional changes (2, 3). During match play, approximately 20% of sprints involve COD (4), highlighting the regular incorporation of directional changes within high-intensity locomotor activities. Moreover, COD movements in basketball often involve high acceleration (5). In both offensive and defensive situations, players who excel in COD are likely to gain spatial advantages on court. Therefore, COD ability is regarded as a critical physical attribute that strength and conditioning (S&C) coaches should prioritize when designing training programs for basketball athletes (6).

Triaxial force plates are commonly used to quantify COD kinetics/kinematics (7, 8). Specifically, ground reaction forces (GRFs) measured by force plates enable the estimation of center of mass (COM) acceleration based on Newtonian mechanics using GRF relative to body mass (GRF_rel_) (9). Among the various kinetic outputs, the horizontal GRF_rel_ has been identified as a primary mechanical component associated with COD performance during 180° turns and lateral shuffles (8, 10, 11). Although triaxial force plates allow precise estimation of COM acceleration from GRFs, their use is generally restricted to laboratory settings, as they cannot be embedded across the court. Traditionally, COD performance has been assessed in field settings using task completion time, providing a simple and practical evaluation metric. However, such time-based assessments are influenced by multiple factors, including linear sprint speed, and may not accurately isolate the mechanical contributors to COD task outcomes (12–14). Therefore, alternative methods that capture the horizontal COM acceleration during COD maneuvers are increasingly needed.

In recent years, triaxial inertial measurement units (IMUs)—which incorporate accelerometers, gyroscopes, and magnetometers—have been increasingly used in applied sports settings. Resultant acceleration derived from IMUs (ACC_IMU_) has been employed as an indicator of external load during high-intensity activities such as COD and sprinting (3), particularly for in-season load monitoring (15). Resultant ACC_IMU_ has been highlighted as a practical metric for quantifying the mechanical demands of rapid movement (5, 16). For instance, Koyama et al. (5) reported that elite basketball players executed approximately 400 COD actions per game with resultant ACC_IMU_ exceeding 4*g* (1*g* = 9.81 m/s^2^), underscoring its potential as a proxy for the mechanical demands of COD movements. Moreover, these integrated sensors can theoretically correct for orientation, enabling accurate evaluation of both vertical and horizontal ACC_IMU_ (17). Previous research has demonstrated that peak vertical ACC_IMU_ accurately estimates vertical GRF during jumping tasks when sensors are positioned near the COM (18). Gurchiek et al. further demonstrated that the step-averaged resultant GRF estimated from ACC_IMU_ was valid during COD tasks (19). However, other studies have reported that peak resultant and horizontal ACC_IMU_ exhibit overestimation, including both fixed and proportional biases, during COD tasks (20, 21). Notably, Roell et al. included multiple movement types within their analyses, which may have contributed to increased error and variability in horizontal acceleration (20). Although those studies examined non-athlete participants (19, 21), systematic bias may be even greater in elite athletes who are capable of generating higher accelerative forces.

Therefore, this study aimed to examine the relationship between horizontal GRF_rel_ and ACC_IMU_ measures during 180° COD tasks. Furthermore, we examined the pattern of differences (fixed and/or proportional bias) between the GRF_rel_ and ACC_IMU_ measures. We hypothesized that horizontal GRF_rel_ would be significantly correlated with both horizontal and resultant ACC_IMU_, but that ACC-based estimates would exhibit overestimation due to the presence of fixed and/or proportional bias.

## 2 Methods

### 2.1 Participants

Fourteen female basketball players (age: 24.4 ± 4.5 years, range: 18–27 years; height: 174.5 ± 7.1 cm; body mass: 68.5 ± 10.8 kg) who participated in the Japanese Women's National Basketball Team training camp took part in this study. According to the athlete classification by McKay et al. (22), 10 and four players were classified as Tier 5 (World Class) and Tier 4 (Elite/International Level), respectively. All participants were free from injuries that might limit their physical performance and were unrestricted in practice.

This study was approved by the Institutional Ethics Committee of the Japan Institute of Sports Sciences (no. 2021-057-3). All tests were conducted at the beginning of a training session during the national training camp as part of the team assessment. Prior to testing, all participants were informed of the potential benefits and risks of the test, and written consent was obtained regarding the potential use of their data for research purposes. Information on the study's purpose and the option to opt out were made publicly available on the Japan High Performance Sports Center website (https://www.jpnsport.go.jp/hpsc/business/ourwork/tabid/1322/Default.aspx), allowing athletes to opt out without facing any disadvantages.

### 2.2 Procedures

**Figure 1:**
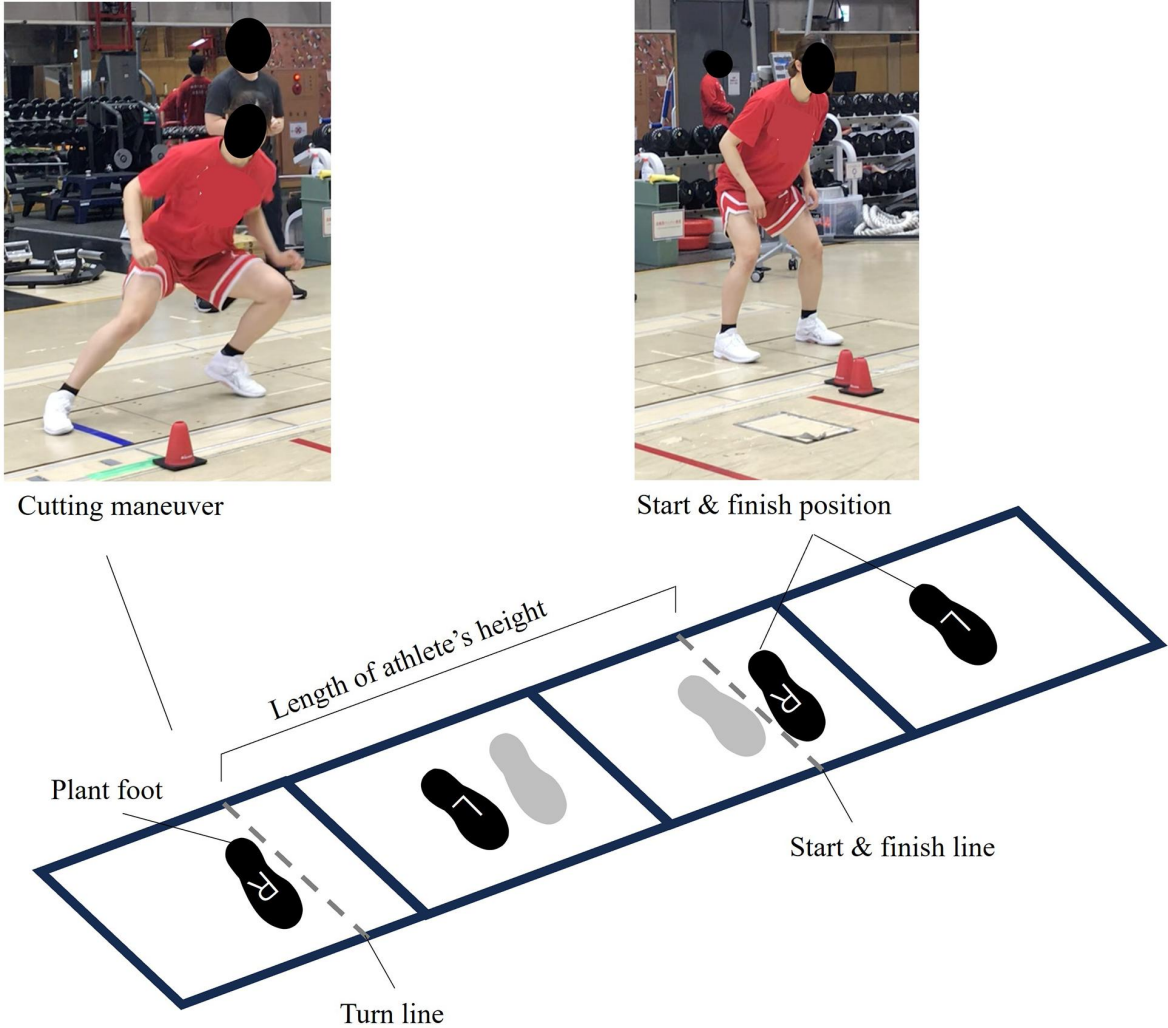
Experimental setup for the lateral shuffle task. This figure illustrates a representative starting foot position and foot placement for COD, with the right foot acting as the plant foot. The gray footprints represent the first step.

**Figure 2:**
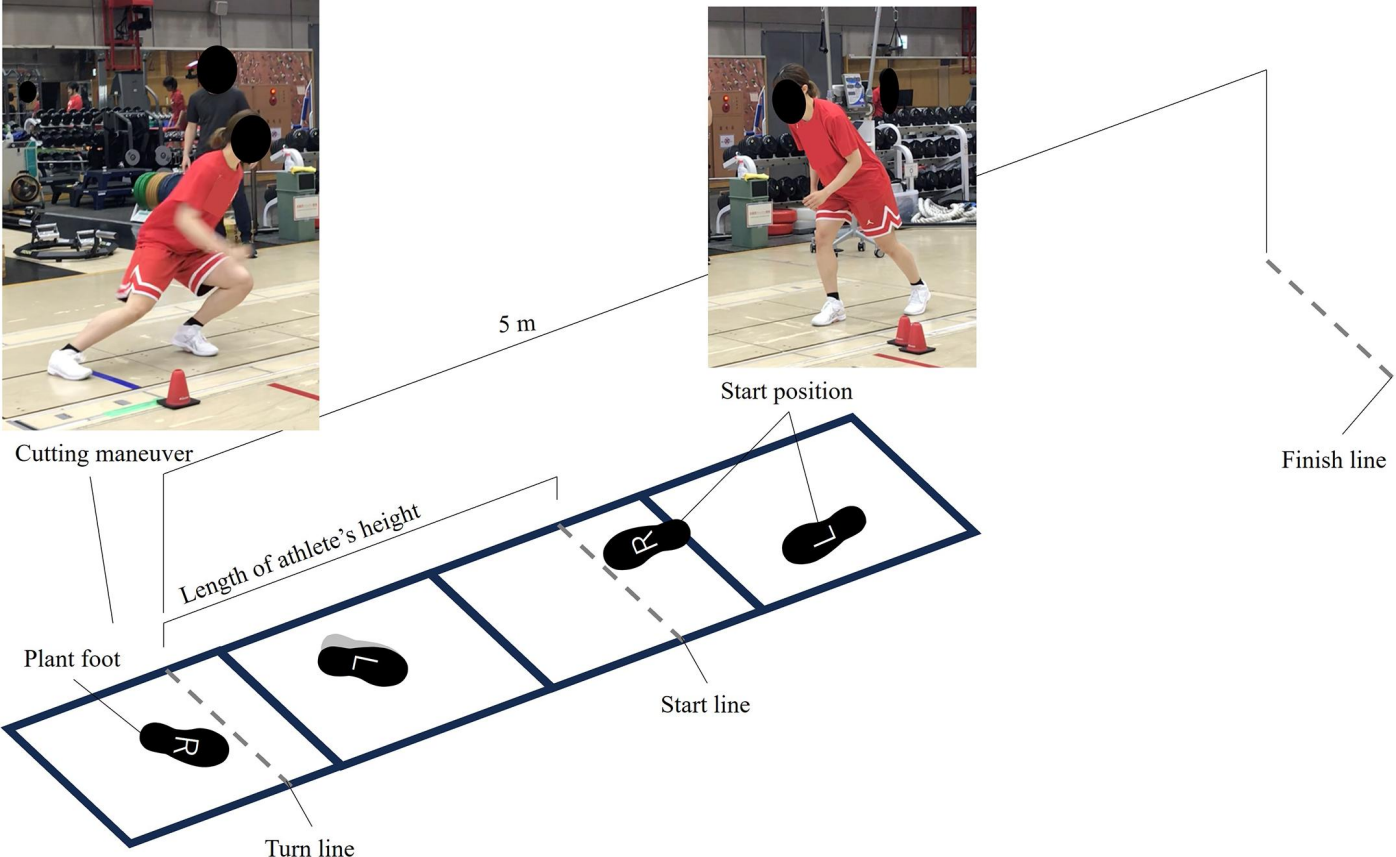
Experimental setup for the 180° turn task. This figure illustrates a representative starting foot position and foot placement for COD, with the right foot acting as the plant foot.

The participants wore their usual training apparel and basketball shoes. Following an S&C coach-led dynamic warm-up and an explanation of the test protocols, they performed two to three submaximal familiarization trials for each task. Each participant performed two 180° COD tasks with maximal effort on four consecutive force plates (Type 9281EA, 0.9 m × 0.6 m, Kistler, Winterthur, Switzerland): a lateral shuffle (Figure 1) followed by a 180° turn (Figure 2). For each task, two consecutive trials were performed with each plant foot (left and right), and the order of the plant-foot conditions was self-selected, resulting in a total of eight trials per participant. The starting and directional change points were marked on the plates, and the distance between them was adjusted based on each participant's height (11).

In the lateral shuffle (Figure 1), athletes began in an athletic stance with the outer edge of the plant foot just behind the starting line. They performed a two-step lateral shuffle to a designated line, cut, and returned to the starting position (11, 23). In the 180° turn (Figure 2), participants began in a staggered stance with the toe of the plant foot just behind the starting line. They sprinted forward in two steps to a designated line, planted the second step beyond it to execute a 180° turn, and then sprinted back to a finish line 5 m from the point of direction change.

Each trial was initiated by a verbal cue from the experimenter, and participants were required to maintain their starting stance for at least 1 s beforehand while standing on the force plates (see Figure 1). If a participant slipped, turned before crossing the designated turn line, or failed to step completely on the force plates, the trial was discarded and repeated after adequate rest.

The IMU device (100 Hz, KINEXON GmbH, Munich, Germany; 47 mm × 33 mm × 7.5 mm, 15 g), which includes a triaxial accelerometer (±16 g), triaxial gyroscope (±4,000 deg/s), and triaxial magnetometer (±16 *μ*T), was securely mounted centrally at the level of the sacrum using a specially designed pouch with a clip attached to a waistband of the shorts. Although the manufacturer recommends placement above the right posterior superior iliac spine during routine monitoring in training and matches (24), we selected a midline sacral placement as a proxy location for the COM (18, 19) to minimize left–right directional bias during COD tasks. The device is part of a commercially available multi-unit team monitoring system with integrated analytics and is widely employed for load management in indoor team sports (24, 25).

### 2.3 Data analyses

These COD tasks were performed using the same procedures as in our previous study (23). During the COD tasks, the GRF was calculated as the sum of the bilateral values, representing the external forces acting on the body during ground contact, along with the force of gravity. The GRFs were smoothed using a fourth-order low-pass Butterworth filter with a cutoff frequency of 25 Hz, in accordance with previous COD studies evaluating peak and average GRF (8, 26). The GRF data were resampled at 100 Hz to match the IMU sampling frequency. Body weight was calculated as the 0.5 s moving average of the vertical GRF with the smallest standard deviation (SD) during the starting stance (27). Each window was shifted by one frame across the starting stance phase, and the body weight corresponding to the window with the smallest SD was used for the analysis. We confirmed that the coefficient of variation of the vertical GRF during the window in each trial was low (less than 1.5%).

The triaxial acceleration time-series data were exported from the IMU system as orientation-corrected acceleration signals (ACC_IMU_) in CSV format. These signals were subsequently low-pass filtered using a zero-phase 4th-order Butterworth filter. The cutoff frequency was determined via Winter's residual analysis (9) and fixed at 25 Hz to standardize the analysis while preserving the step-to-step acceleration–deceleration characteristics during the 180° COD tasks. This procedure is consistent with established IMU practice employing residual analysis (17, 20). The resultant ACC_IMU_ (vector norm of *x*, *y* and *z* axes) was then calculated in Microsoft Excel (Microsoft Corp., Redmond, WA, USA). In the starting position, the vertical ACC_IMU_ was approximately 1, consistent with alignment to gravity. Although the manufacturer has not disclosed the specific sensor-fusion algorithms used, the exported ACC_IMU_ can therefore be regarded as orientation-corrected acceleration (20).

The ACC_IMU_ was expressed as a dimensionless value relative to gravitational acceleration. According to Newton's Second Law (***F*** = *m**a***), COM acceleration is governed by the net external force acting on the body (i.e., GRF and gravity) scaled by body mass. The GRF was normalized to body weight (GRF_rel_ in N/N) to yield a dimensionless value, thereby facilitating comparison between two dimensionless quantities, ACC_IMU_ and GRF_rel_ (21). The horizontal GRF_rel_ was treated as the braking/propulsive component, following Dos'Santos et al. (8), with the orthogonal horizontal component considered negligible. In contrast, the horizontal ACC_IMU_ included both the lateral and fore–aft components (e.g., resulting from pelvic rotation). Peak horizontal and resultant ACC_IMU_ during plant foot contact were identified by time-aligning the ACC_IMU_ data with the GRF waveform and confirming approximate time frames (e.g., quiet standing, movement onset, turn) using video recordings (iPad Pro; Apple Inc., Cupertino, CA, USA). Although some previous studies have used mean GRF and other force–time metrics as indicators of COD performance (8, 23), GRF and ACC_IMU_ time series were not hardware-synchronized in the present study. Therefore, analyses were restricted to peak metrics rather than impulse- or force–time–derived measures. The GRF calculations and IMU filtering were performed using MATLAB 2019b (MathWorks, Inc., Natick, MA, USA).

### 2.4 Statistical analysis

Statistical analyses were performed using IBM SPSS Statistics (version 30.0.0.0; IBM Inc., Armonk, NY, USA), with the significance level set at *p* < 0.05. The normality of each variable was assessed using the Shapiro–Wilk test and *Q*–*Q* plots. All variables were deemed approximately normally distributed, as indicated by the Shapiro–Wilk test (*p* ≥ 0.05) and/or the *Q*–*Q* plot exhibiting an approximately linear pattern with only minor deviations. Accordingly, all data are presented as mean ± SD. Intrasession reliability across the two trials was evaluated using the intraclass correlation coefficient (ICC_2,2_) and the coefficient of variation (CV) for the peak horizontal GRF_rel_, peak resultant ACC_IMU_, and peak horizontal ACC_IMU_. The ICC_2,2_ values were interpreted according to the following thresholds: 0.1–0.29 = low, 0.3–0.49 = moderate, 0.5–0.69 = high, 0.7–0.89 = very high, 0.9 = nearly perfect, and 1.0 = perfect (28). CV% was interpreted according to the following thresholds: <5% = small, 5%–20% = moderate, and >20% = large (21).

Paired *t*-tests were conducted to compare peak horizontal GRF_rel_ with both peak resultant ACC_IMU_ and peak horizontal ACC_IMU_ using the mean of two trials. The magnitude of the differences was evaluated using Cohen's *d*, with interpretation (28, 29).

Ordinary least products (OLP, Model II) regression was employed to assess fixed and proportional bias when comparing peak ACC_IMU_ (Y) with peak horizontal GRF_rel_ (X), in accordance with the methodology of a previous study (30). The 95% confidence intervals (CIs) for the OLP slope and intercept were obtained using bias-corrected and accelerated (BCa) bootstrap resampling with 2,000 iterations. Proportional bias was inferred when the 95% CI for the slope excluded 1.0, whereas fixed bias was identified when the 95% CI for the intercept excluded 0 (31). Pearson's product–moment correlation coefficients were calculated to assess the associations between peak horizontal GRF_rel_ and both peak resultant ACC_IMU_ and peak horizontal ACC_IMU_ based on the mean of the two trials. Correlation strength was classified as follows: 0.10–0.29 = small, 0.30–0.49 = moderate, 0.50–0.69 = large, 0.70–0.89 = very large, 0.90–0.99 = almost perfect, and 1.0 = perfect (29).

## 3 Results

Descriptive statistics and intrasession reliability are shown in Table 1. Most variables demonstrated very high intrasession reliability (ICC_2,2_ > 0.7) with small-to-moderate CV% values (3.85–12.74%), except for peak horizontal ACC_IMU_ during the 180° turn with the right plant foot (ICC_2,2_ = 0.67, high; CV% = 12.74%, moderate) (Table 1).

**Table 1:** Descriptive statistics and between-trial reliability for 180° COD tests.

Variables	Task	Foot	First trial	Second trial	ICC_2,2_	CV%
			Mean ± SD	Mean ± SD		
Peak horizontal GRF_rel_ (dimensionless)	Lateral shuffle	R	1.90 ± 0.30	1.85 ± 0.24	0.73	6.22
		L	1.92 ± 0.36	1.93 ± 0.28	0.81	7.72
	180° turn	R	2.09 ± 0.30	2.20 ± 0.32	0.95	3.85
		L	2.06 ± 0.29	2.21 ± 0.32	0.82	6.43
Peak resultant ACC_IMU_ (dimensionless)	Lateral shuffle	R	5.12 ± 0.93	5.10 ± 1.40	0.88	10.08
		L	5.35 ± 1.43	5.44 ± 1.45	0.87	10.55
	180° turn	R	5.03 ± 0.59	5.45 ± 1.12	0.72	8.46
		L	5.81 ± 1.08	6.27 ± 1.23	0.85	7.68
Peak horizontal ACC_IMU_ (dimensionless)	Lateral shuffle	R	4.89 ± 0.85	4.84 ± 1.41	0.86	11.10
		L	4.77 ± 1.63	4.86 ± 1.63	0.87	12.74
	180° turn	R	4.41 ± 0.58	4.77 ± 1.31	0.67	12.74
		L	4.74 ± 1.37	5.40 ± 1.48	0.85	12.58

GRF_rel_, ground reaction force normalized by body weight; calculated as the sum of bilateral GRFs during COD tasks. Peak GRF values denote the maximum of the sample-by-sample sum of the right and left feet. ACC_IMU_, sacrum-mounted IMU acceleration expressed as a dimensionless value relative to gravitational acceleration. “R” and “L” indicate the right and left plant foot during cutting, respectively.

The peak horizontal ACC_IMU_ was significantly higher than the peak horizontal GRF_rel_ during both lateral shuffles (right: *p* < 0.001, *d* = 0.94; left: *p* < 0.001, *d* = 1.39) and 180° turns (right: *p* < 0.001, *d* = 0.83; left: *p* < 0.001, *d* = 1.24) (Table 2).

**Table 2:** Difference between peak horizontal GRF_rel_ and peak horizontal ACC_IMU_.

Task	Foot	Peak horizontal GRF_rel_ (dimensionless)	Peak horizontal ACC_IMU_ (dimensionless)	*p*	*d*
		Mean ± SD	Mean ± SD		
Lateral shuffle	R	1.87 ± 0.24	4.86 ± 1.08	<0.001	0.94
	L	1.92 ± 0.29	4.81 ± 1.53	<0.001	1.39
180° turn	R	2.14 ± 0.31	4.59 ± 0.88	<0.001	0.83
	L	2.14 ± 0.29	5.07 ± 1.35	<0.001	1.24

GRF, ground reaction force; ACC_IMU_, sacrum-mounted IMU acceleration expressed as a dimensionless value relative to gravitational acceleration. Peak GRF values denote the maximum of the sample-by-sample sum of forces from the right and left feet. “R” and “L” indicate the right and left plant foot during cutting, respectively.

In the lateral shuffle, the peak horizontal GRF_rel_ was significantly correlated with the peak horizontal ACC_IMU_ (right: *r* = 0.69, *p* = 0.006; left: *r* = 0.55, *p* = 0.041) (Table 3, Supplementary Figure 1). OLP regression indicated proportional and fixed bias (slope 95% CIs: 2.75–6.50, right; 2.35–7.77, left; intercept 95% CIs: −7.12 to −0.66, right; −11.12 to −1.05, left). Similarly, the peak horizontal GRF_rel_ demonstrated significant positive correlations with the peak resultant ACC_IMU_ (right: *r* = 0.68, *p* = 0.007; left: *r* = 0.72, *p* = 0.003). Concordantly, OLP regression also indicated proportional and fixed bias (slope 95% CIs: 2.86–7.10, right; 2.75–7.04, left; intercept 95% CIs: −7.94 to −0.47, right; −8.57 to −0.06, left).

**Table 3:** Pearson correlations and OLP regression (Y on X) performed to examine the relationship between peak horizontal GRF_rel_ (X) and IMU-derived peak acceleration (Y: horizontal or resultant).

Comparison	Task	Foot	*r*	*p*	Slope (95% CI)	Intercept (95% CI)
Horizontal GRF_rel_ and horizontal ACC_IMU_	Lateral Shuffle	R	0.69	0.006	4.55 (2.75, 6.50)^†^	−3.65 (−7.12, −0.66)^‡^
		L	0.55	0.041	5.23 (2.35, 7.77)^†^	−5.24 (−11.12, −1.05)^‡^
	180° turn	R	0.33	0.248	2.86 (−2.74, 3.94)	−1.53 (−4.44, 10.21)
		L	0.49	0.073	4.75 (−3.50, 8.50)	−5.07 (−13.80, 11.23)
Horizontal GRF_rel_ and resultant ACC_IMU_	Lateral Shuffle	R	0.68	0.007	4.70 (2.86, 7.10)^†^	−3.68 (−7.94, −0.47)^‡^
		L	0.72	0.003	4.60 (2.75, 7.04)^†^	−3.45 (−8.57, −0.06)^‡^
	180° turn	R	0.68	0.007	2.61 (1.61, 3.42)^†^	−0.35 (−2.25, 1.59)
		L	0.64	0.013	3.84 (1.85, 6.49)^†^	−2.16 (−8.26, 1.93)

GRF, ground reaction force; ACC_IMU_, sacrum-mounted IMU acceleration expressed as a dimensionless value relative to gravitational acceleration. Peak GRF values denote the maximum of the sample-by-sample sum of forces from the right and left feet. “R” and “L” indicate the right and left plant foot during cutting, respectively. OLP, ordinary least products; CI, confidence interval.

^†^ If the 95% confidence interval for the slope does not include 1.0, then proportional bias is present.

^‡^ If the 95% confidence interval for the intercept does not include 0, then fixed bias is present.

In contrast, during the 180° turns, the correlation between the peak horizontal GRF_rel_ and the peak horizontal ACC_IMU_ was weak and not statistically significant (right: *r* = 0.33, *p* = 0.248; left: *r* = 0.49, *p* = 0.073). Consistently, the OLP regression revealed no proportional or fixed bias in these relationships. However, the peak horizontal GRF_rel_ was significantly correlated with the peak resultant ACC_IMU_ (right: *r* = 0.68, *p* = 0.007; left: *r* = 0.64, *p* = 0.013), and OLP regression indicated proportional bias (slope 95% CIs: 1.61–3.42, right; 1.85–6.49, left) without fixed bias in either direction (Table 3, Supplementary Figure 1).

## 4 Discussion

The present study investigated the associations between GRF_rel_ and ACC_IMU_ during 180° COD tasks. In the lateral shuffle task, GRF_rel_ demonstrated significant correlations with both peak horizontal and resultant ACC_IMU_, and both ACC_IMU_ variables exhibited proportional and fixed biases. Conversely, during the 180° turn task, the correlations between peak horizontal GRF_rel_ and peak horizontal ACC_IMU_ were weak, and neither proportional nor fixed bias was observed; however, proportional bias was evident for peak resultant ACC_IMU_. Furthermore, peak horizontal ACC_IMU_ values were significantly greater than peak horizontal GRF_rel_. Most variables exhibited adequate intrasession reliability (ICC_2,2_ > 0.7) with small-to-moderate CV% values (3.85%–12.74%). Collectively, these results support the capability of ACC_IMU_ to reflect horizontal GRF characteristics during 180° COD maneuvers.

Peak horizontal ACC_IMU_ values were significantly greater than GRF_rel_ values for both tasks. The observed overestimation is likely attributable to substantial vertical components present within the peak horizontal ACC_IMU_ signal. Although previous COD studies have reported vertical GRF to be approximately 1.5 times greater than horizontal GRF (8), the present study recorded peak horizontal ACC_IMU_ values of 4.4–4.9 g and peak resultant ACC_IMU_ values of 5.0–6.2 g, indicating notable cross-axis mixing even when peak timings differed slightly. Gurchiek et al. (19) identified direction-dependent systematic errors, with horizontal components being particularly susceptible to bias, while Roell et al. (20) highlighted the algorithmic factors, such as sensor fusion and coordinate transformations, which can amplify such discrepancies. Collectively, the findings suggest that overestimation primarily reflects signal-processing factors. Additionally, given the waistband-mounted pouch fixation, small movements of the pouch/attachment system relative to the pelvis may introduce motion artefact and inflate peak horizontal ACC_IMU_ (32).

During the lateral shuffle task, peak resultant and horizontal ACC_IMU_ were significantly correlated with peak horizontal GRF_rel_. OLP regression further indicated the presence of proportional bias for both acceleration metrics, suggesting that measured ACC_IMU_ values systematically scaled with GRF magnitude. Throughout these movements, the chest and pelvis remained perpendicular to the direction of travel, and the trunk stayed upright in the frontal plane (33, 34). Minimal changes in trunk posture during lateral shuffle movements allow the GRF generated by the plant foot to align more closely with the direction of the sacrum ACC_IMU_, thereby enhancing the GRF_ref_–ACC_IMU_ correlation. Previous studies have shown that a higher peak horizontal GRF_rel_ is associated with superior lateral shuffle performance, as the lateral cutting index is calculated by dividing the velocity of the sacral segment at takeoff by the foot contact time during lateral shuffles (11). These findings indicate that, during lateral shuffle tasks, both peak resultant and peak horizontal ACC_IMU_ may function as practical indicators of peak horizontal GRF_rel_.

Conversely, during the 180° turn, a significant positive correlation and proportional bias were observed between peak resultant ACC_IMU_ and peak horizontal GRF_rel_, whereas peak horizontal ACC_IMU_ exhibited neither a significant correlation nor proportional bias with peak horizontal GRF_rel_. This discrepancy may be explained by differences in movement patterns; during the 180° turn, pronounced pelvic rotation and trunk inclination toward the new direction of travel were commonly observed (8, 26, 35). In these instances, peak horizontal ACC_IMU_ is more susceptible to orientation-correction errors, whereas peak resultant ACC_IMU_—calculated as the vector norm—is less influenced by angular variations and may therefore provide a more reliable measure. For instance, Nakamura et al. (35) reported that the trunk inclination angle in the new direction was approximately 50°, and Dos'Santos et al. (8) demonstrated that lateral trunk leaning and pelvic rotation are key biomechanical determinants of faster performance in 180° turn tasks. These kinematic features likely underlie the mismatch between local acceleration signals captured by IMUs and the actual direction and magnitude of the GRF. Previous research has demonstrated that ACC_IMU_ estimates can be compromised during high-intensity, multi-planar movements when substantial trunk rotation or impact forces are present (19–21). With a single sacrum IMU, such rotational and soft-tissue artifacts may further weaken the horizontal ACC_IMU_–GRF_rel_ coupling, whereas peak resultant ACC_IMU_ remains a more robust and repeatable indicator of integrated COD load. Similar findings were reported by Wundersitz et al., who demonstrated that peak resultant ACC_IMU_ exhibited proportional bias against GRF during COD tasks, leading to a systematic overestimation of mechanical load (21). Therefore, in single-IMU, on-court COD assessments, peak horizontal ACC_IMU_ may be appropriate when trunk posture is relatively constrained (e.g., lateral shuffle), whereas for highly multi-planar tasks (e.g., 180° turn), peak resultant ACC_IMU_ is preferred due to its greater robustness and repeatability. Accordingly, in COD movements characterized by marked trunk rotation and inclination, peak resultant ACC_IMU_, which integrates acceleration across all axes, may better represent the GRF than its horizontal component by more comprehensively capturing the mechanical load imposed during the 180° turn.

Traditionally, COD performance has been assessed using task completion times as a single outcome measure. However, these times often show high correlations with linear sprint ability, thereby potentially masking the true COD ability (12–14). Previous in-game studies utilized an IMU to quantify high-intensity COD events and proposed its utility in applied contexts (5, 16). For instance, Alanen et al. (36) proposed that IMU could enable COD-specific assessments in ecologically valid contexts. In this context, our findings suggest that IMU-derived acceleration metrics, such as peak ACC_IMU_, provide insight into the instantaneous COM acceleration profile during COD maneuvers, rather than relying solely on a single time outcome.

This study has several limitations. First, the cohort comprised fourteen world-class and international-level female basketball players, a small and homogeneous sample. Accordingly, the magnitudes of forces and accelerations, as well as correlation strength, may differ across male or sub-elite cohorts and in other sports due to differences in approach speed, technique, and anthropometrics. Thus, our conclusions should not be over-generalized beyond similar populations. Second, variations in COD angle may also yield different outcomes. Third, the IMU was secured to participants’ clothing via a specialized sleeve and clip, making complete elimination of sensor displacement challenging. This may partly explain the lower intrasession reliability observed for peak horizontal acceleration in the right-foot 180° turn, where rapid pelvic rotation and high accelerations may increase small movements of the IMU relative to the pelvis. The sensor location also diverged from the manufacturer's recommendation, raising questions about whether lateral movements were captured equally on both sides. Fourth, because the force-plate and IMU time series were aligned *post hoc* (i.e., without hardware synchronization), we restricted our analyses to peak metrics; this precluded robust comparisons of impulse-, contact-time-, and force–time–derived measures. Finally, the choice of cutoff frequency for data smoothing could also have influenced the results; however, this factor was not investigated in detail because it is beyond the scope of the present study (20, 37). These limitations warrant further investigation. Nevertheless, the capacity to quantify COD ability outside the laboratory, in practice and competition, offers substantial value despite such constraints.

## 5 Conclusion

This study demonstrated that ACC_IMU_ measured at the sacrum was associated with peak horizontal GRF_rel_ during 180° COD tasks in elite female basketball players. The pattern of these relationships differed by task, with peak horizontal ACC_IMU_ aligning more closely with GRF_rel_ during the lateral shuffle, whereas peak resultant ACC_IMU_ showed a larger association with GRF_rel_ during the 180° turn. These findings indicate that ACC_IMU_ captures task-specific mechanical characteristics of COD maneuvers and support its use for field-based COD assessment.

## Data Availability

The raw data supporting the conclusions of this article will be made available by the authors, without undue reservation.
