# Supplementary material for: Relationship between ground reaction force and sacrum acceleration during 180° change of direction maneuvers in elite female basketball players
Source: Front Sports Act Living. 2026 Feb 12;8:1665797. doi: 10.3389/fspor.2026.1665797 (PMC12935448; doi:10.3389/fspor.2026.1665797)
Supplement: Supplementary file 1 [file Image1.pdf]

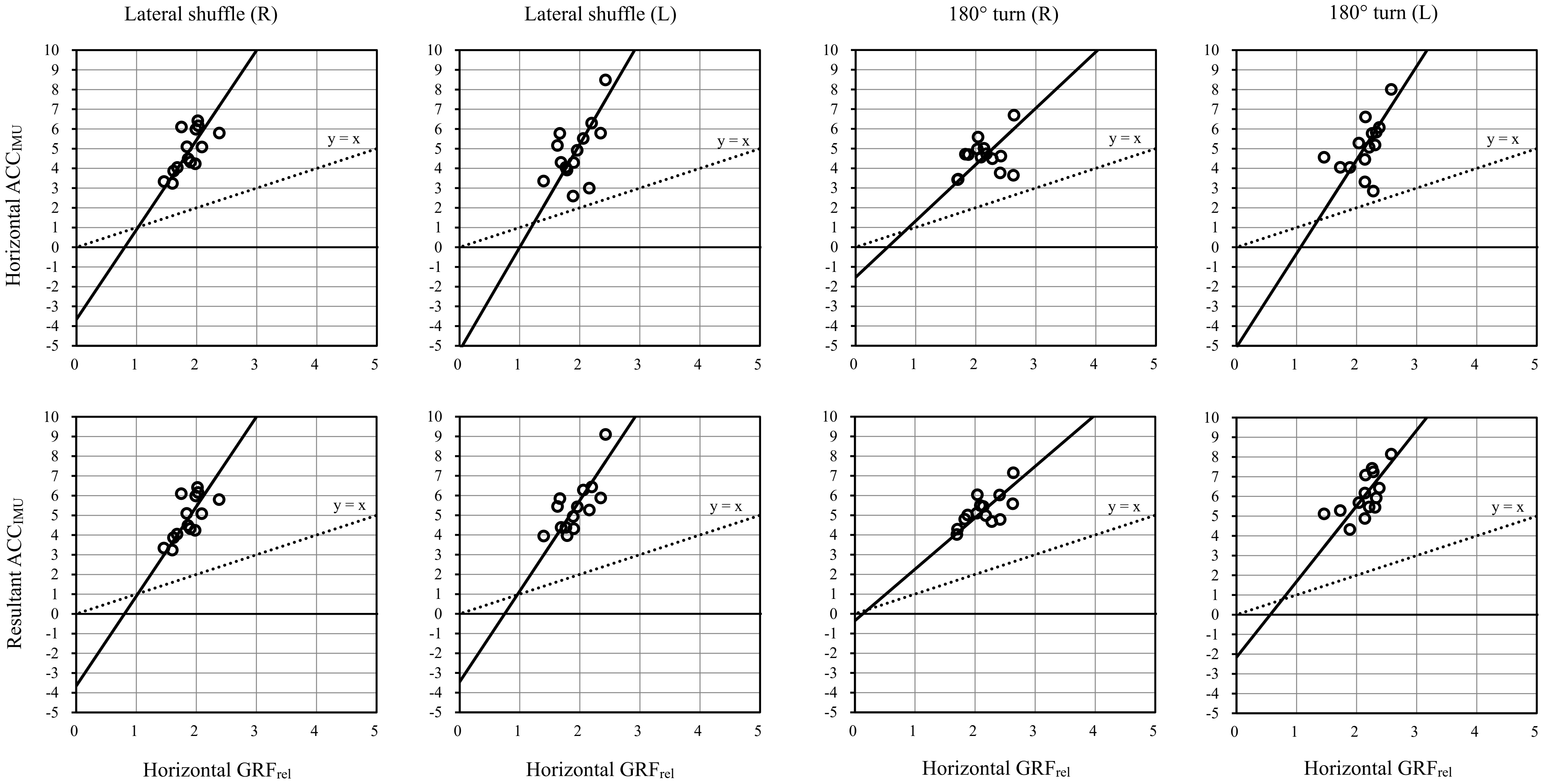

Supplementary Figure 1. Ordinary least products (OLP, Model II) regression analysis comparing peak accelerations (ACC) derived from the IMU (Y-axis) with peak horizontal ground reaction forces normalized to body weight (GRF$_{rel}$; X-axis). The solid line represents the OLP regression line, and the dashed line indicates the line of identity (Y = X).
